# Meraculous: *De Novo* Genome Assembly with Short Paired-End Reads

**DOI:** 10.1371/journal.pone.0023501

**Published:** 2011-08-18

**Authors:** Jarrod A. Chapman, Isaac Ho, Sirisha Sunkara, Shujun Luo, Gary P. Schroth, Daniel S. Rokhsar

**Affiliations:** 1 U.S. Department of Energy Joint Genome Institute, Walnut Creek, California, United States of America; 2 Illumina, Inc., Hayward, California, United States of America; 3 Department of Molecular and Cell Biology, University of California, Berkeley, California, United States of America; Johns Hopkins University, United States of America

## Abstract

We describe a new algorithm, meraculous, for whole genome assembly of deep paired-end short reads, and apply it to the assembly of a dataset of paired 75-bp Illumina reads derived from the 15.4 megabase genome of the haploid yeast *Pichia stipitis*. More than 95% of the genome is recovered, with no errors; half the assembled sequence is in contigs longer than 101 kilobases and in scaffolds longer than 269 kilobases. Incorporating fosmid ends recovers entire chromosomes. Meraculous relies on an efficient and conservative traversal of the subgraph of the *k*-mer (deBruijn) graph of oligonucleotides with unique high quality extensions in the dataset, avoiding an explicit error correction step as used in other short-read assemblers. A novel memory-efficient hashing scheme is introduced. The resulting contigs are ordered and oriented using paired reads separated by ∼280 bp or ∼3.2 kbp, and many gaps between contigs can be closed using paired-end placements. Practical issues with the dataset are described, and prospects for assembling larger genomes are discussed.

## Introduction

Massively parallel sequencing methods introduced over the past few years provide cost-effective, highly redundant sampling of genomes (reviewed in [1]). Pyrosequencing reads are approaching conventional dideoxy capillary sequences in their read length, providing a direct substitute for Sanger sequences [2]. While sequencing by synthesis produces substantially shorter reads, it has lower cost per base and higher throughput [3]. Such data has proven useful for re-sequencing variant genomes [4], [5], [6], since short reads can be readily aligned to a reference, and the error rates are low enough that variation can be detected by consistent discrepancy of the aligned short reads versus the reference. The usefulness of such short-read datasets for *de novo* genome assembly has been the subject of increasing excitement (reviewed in [7] [8]), including recent assemblies of mammalian genomes [9], [10], [11], [12].

Critical to the assembly of short (<100 bp) reads is the use of paired-end sequencing protocols, which were first introduced in the early 1990s for use with Sanger sequencing [13], [14], [15]. The importance of using a range of paired-end linkages to organize non-repetitive contigs into scaffolds by linking over repetitive regions was presciently emphasized by Weber and Myers [16] in the context of human whole genome shotgun sequencing. This approach became the dominant paradigm for genome sequencing in the last decade. Pairing also allows the assembly of localized regions that are repetitive on the scale of the entire genome, since reads that derive from a particular localized copy of a repeat can often be inferred by the placement of their mate-pair reads in flanking unique sequences. With short reads the advantages of paired-end approaches are accentuated [17], and this strategy figures prominently in recently developed short-read assemblers (reviewed in ref. [18]) including EULER-SR [19], Velvet [20], [21], ALLPATHS [22], [23], ABySS [9] and SOAPdenovo [11]. These assemblers all take advantage of the deBruijn graph representation of the assembly problem [24], in which reads are decomposed into overlapping words of length *k* (“*k*-mers”), where *k* is a fraction of the read length.

Here we present a new assembler, called meraculous, that relies on an efficient and conservative traversal of a subgraph of the k-mer (deBruijn) graph of oligonucleotides with unique high quality extensions in the dataset. Unlike other short-read assemblers, meraculous avoids an explicit error correction step, instead relying on base quality scores. Meraculous also incorporates a novel low-memory hash structure to access the deBruijn graph, allowing a small memory footprint compared with other short-read assemblers. To test meraculous we also report here a deep Illumina dataset for a yeast genome.

*Pichia stipitis* CBS 6054 is a predominantly haploid yeast that efficiently produces ethanol from xylose and other polysaccharides [25]. The *P. stipitis* genome (N = 8; GC = 41.1%) was previously sequenced and finished using Sanger methods [26], and has been used to assess the abilities of different next generation sequencing methods to detect variation [6]. As a test set for meraculous, we report a dataset of three lanes of 75 bp paired-end shotgun for *P. stipitis* produced using Illumina sequencing-by-synthesis methods, with both short-range (∼280 bp) and medium-range (∼3.2 kbp) pairing data. These data provide a nominal 425-fold redundant sampling of the 15.4 million base pair (Mbp) genome. The meraculous assembly reconstructs 95% of the *Pichia* genome in long contigs and scaffolds without any errors. If we use the standard “N50” measure, half the genome is in contigs longer than 101 kbp and scaffolds longer than 269 kbp. Adding a modest number of fosmid ends recovered entire chromosomes. Many stages of the meraculous algorithm are parallelized, and to document their scalability we describe an assembly of simulated data for the ∼120 Mbp *Arabidopsis thaliana* genome, and show that for mammalian genomes the limiting memory structure requires less than 10 Gb of RAM.

The meraculous software, *Pichia* shotgun sequence and assembly is available for download at ftp://ftp.jgi-psf.org/pub/JGI_data/meraculous/.

## Materials and Methods

### *Pichia* shotgun sequencing

**Figure 1:**
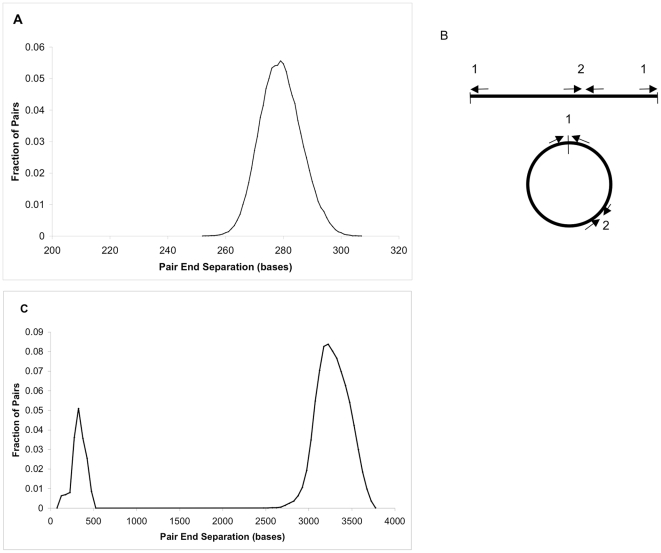
Paired ends. **A. Fragment pair end separation distribution.** Pairs are separated by 279±7 bp. **B. Mate-pairs are produced by circularizing a genomic segment** (vertical line indicates junction). End-sequences from sheared fragments that contain the junction (1) represent reads that point outward at the ends of the original segment. End-sequences from sheared fragments that do not contain the junction (2) are inwardly directed and adjacent on the original segment. **C. Mate-pair end separation distribution.** Two-thirds of all pairs are found to be divergently oriented and separated by 3.2±0.2 kb. An artifactual population of convergently oriented pairs separated by less than 500 bp is apparent, representing fragments of type (2) shown above in panel B.

We constructed short insert “fragment” paired-end libraries, with an average insert size of ∼300 bp, using “Paired-End DNA Sample Prep Kit V1,” Catalog # PE-102-1001, from Illumina (San Diego, CA). We also constructed longer-range “mate pair” or “jumping” libraries, with an average insert size of ∼3 kbp, using Illumina's “Mate Pair Library Prep Kit”, Catalog #: PE-112-1002 (**Figure 1**). Both the fragment and mate pair libraries were sequenced at read lengths of 75 bases from both ends (2×75) using the Illumina Genome Analyzer II following manufacture's recommended protocols. Genomic DNA came from the same sample that was used in the earlier Sanger sequencing project [26]. For the fragment library, two channels were sequenced, with 15.5 and 15.7 million clusters reporting sequence. For the jumping library, one channel was sequenced with 12.4 clusters reporting sequence. These reads yield a nominal 425× coverage of the *P. stipitis* genome.

### Pichia reference sequence

The finished *P. stipitis* CBS 6054 genome sequence [26] is NCBI project number NZ_AAVQ01000000, and consists of sequences AAVQ01000001–AAVQ01000002.

### *E. coli* shotgun sequence and reference

A publicly available paired 36 bp Illumina dataset for *E. coli* K-12 MG1655 dataset was downloaded from the NCBI short read archive, project SRX000429. The finished reference sequence for this strain [27] is Genbank sequence gi|48994873|gb|U00096.2.

### Simulated Arabidopsis dataset

A simulated 100× fragment paired-end dataset with realistic error profiles was produced using persimmonator (Bret Barnes, Illumina). Insert sizes were normally distributed with mean 300 bp and standard deviation 30 bp. Dataset is available upon request.

### Assembly algorithm

The algorithm is encoded in four modules encoded in Perl as described below.

**Figure 2:**
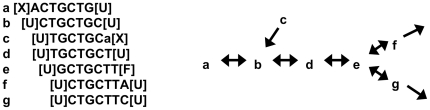
Example of a 7-mer graph. The node **a** is X-terminated to the left. The non-reciprocal linkage between nodes **b** and **c** is removed because the terminal base (lower case “a” in the sequence) of node **c** is low quality. Node **e** is F-terminated to the right. The resultant U-U contig is the union of nodes **b** and **d**: CTGCTGCT.

**Selection of k-mer set.** The shotgun reads are initially processed as follows.
  1. Select an odd integer *k* such that (1) a substantial fraction of the sequence targeted for assembly is unique as *k*-mers, and (2) most reads have multiple overlapping error-free *k*-mers. A *k*-mer is an oligonucleotide sequence of length *k*. For *Pichia* we use *k* = 41.
  2. Count the number of occurrences (multiplicity) of each k-mer in the dataset. This can be accomplished with a single pass through the read set, and for large datasets is readily parallelized by dividing *k*-mers into 4*^m^* bins based on their initial *m* nucleotides, counting *k*-mers in each bin independently. In practice, 16-way parallelization is convenient (*m* = 2).
  3. Choose a threshold multiplicity 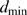 that separates *k*-mers that are likely to contain sequence errors (multiplicity<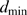) from those that are likely to be error free and occur in the genome (multiplicity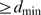). Practically, this threshold should be selected at (or below) the first minimum in the multiplicity curve [28]. We describe below and in Supplemental Text S1 alternate methods for setting 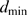. For *Pichia* we use 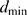 = 10.
  4. Keep only k-mers of multiplicity 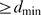 (the “*k*-mer set” below). That is, for the construction of U-U-contigs (see below), ignore *k*-mers of multiplicity less than 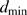 as arising either from sequencing errors or low coverage regions. (*k*-mers with multiplicity below 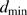 can be recovered in the assembly if they are the unique closure of a gap, see below.)
**meraculous.pl.** meraculous.pl implements the following algorithm, which produces a set of maximal linear sub-paths of the deBruijn graph.
  1. For each *k*-mer, count all single-base extensions (forward and backward) of high quality, that is, occurrences of the k-mer in reads such that the next or previous base has quality value greater than or equal to a threshold (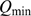) that occur in the shotgun reads. Based on analysis of available data, we use 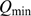 = 20, where *Q* is the quality value assigned to a nucleotide by the Illumina base-calling software. Single base extensions to a base with *Q*>*Q*_min_ are referred to as “high quality extensions” below.
  2. Designate each end of a *k*-mer as X, U, or F depending on whether that end has 0, 1, or 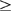
2 distinct high quality extensions of multiplicity at least 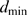. *k*-mer ends designated “X” have no high quality extensions; this condition occurs at persistently unsequenceable or low depth positions. *k*-mer ends marked “U” have a unique high quality extension in the dataset. *k*-mer ends marked “F” represent a “fork” in the deBruijn graph that correspond to exits from a repetitive sequence into multiple alternate sequence contexts. (Polymorphisms in diploid genomes also lead to forks; such cases are not considered further here.)
  3. Store *k*-mers with unique high quality extensions at both ends (*i.e.*, those designated U-U in the previous step) in a hash where the “key” is the *k*-mer and the “value” is a two-letter code [acgt][acgt] that indicates the unique bases that immediately precede and follow the *k*-mer in the read dataset. This hash represents the “U-U graph,” which is a subgraph of the full deBruijn graph. Implementation of a novel hashing scheme is described in more detail below.
  4. Remove all linkages that are not reciprocal. That is, if the *k*-mer *v* is the unique high quality extension of *u* in one direction, then *u* must be the unique high quality extension of *v* in the opposite direction. This step eliminates subpaths corresponding to residual errors (see **Figure 2**) that evade the minimum depth condition.
  5. Arbitrarily select *k*-mers to seed forward and reverse traversals of the U-U graph to produce an initial set of “contigs.” These U-U contigs have the property that each *k*-mer is represented only once in them. The resulting contigs are independent of the selection of seed *k*-mers. We retain only contigs longer than a specifiable minimum length (which is required to exceed 2*k*−1 bases); for the reported *Pichia* assembly, only contigs 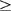
100 bp are considered.
**blastMap.pl.** blastMap.pl aligns reads back to the assembly to identify read-pair information that may be used to link strings of contigs together into scaffolds.
  1. All reads are aligned to the contigs produced by meraculous using BLAST [29]. Aligners designed specifically for short reads could also be used; we initially opted for BLAST for simplicity. Parameters for BLASTN were -b 100 -v 100 -K 100 -e 1e-10 -U -F F -W *k*. Notably the word size was chosen to be *k*, since by construction the U-U contigs contain each U-U *k*-mer exactly once.
  2. Alignments were parsed using a custom Perl script (blastView3.pl, Chapman, unpublished) that reports the highest-scoring HSP (high-scoring segment pair) for all contigs to which a given read is aligned. Alignments of a minimal length (a parameter value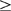 *k*) are retained. For “jumping” libraries, alignment orientations are reversed to conform to standard paired end conventions (see **Figure 1B**), and alignments with less than 600 bp between the 5′ end of the aligning read and a contig end are rejected to prevent inclusion of artifactual pairs which can comprise a significant fraction of these libraries (see Results).
  3. Read vs. contig alignments are categorized as full-length, gap-projecting (alignment ends at contig boundary), incomplete (less than 5 bp unaligned; not at contig boundary), or truncated (at least 5 bp not aligned; not at contig boundary) at each end and also categorized as “pointing out” (3′ end within 1.2× insert size of a contig end), “pointing in” (5′ end within 1.2× insert size of a contig end), or “in the middle” (neither end within 1.2× insert size of a contig end) of the target (contig) sequence.
  4. Full length alignments in which both ends of a pair are placed within a common contig (and appropriately oriented) are used to estimate the insert size of the pair library.
  5. Alignments that project into a gap (at either 3′ or 5′ end) or are “pointing out” from a contig end are retained and categorized as anchored completely within a contig (neither end terminates at a contig boundary), pointing into a gap (3′ end terminates at contig boundary), pointing out of a gap (5′ end terminates at contig boundary), or “splinting” a gap (*i.e.*, having two alignments to different contigs, each of which terminates at a contig boundary). Pairs and singleton reads with these properties are reported for use by subsequent scaffolding and gap-closure steps (discussed below).
**oNo.pl.** oNo.pl uses paired reads and splinting singletons from blastMap to produce a scaffolding by “ordering and orienting” a set of contigs (or a previous scaffolding).
  1. The number of links between contig-end pairs are tabulated and the estimated gap size between contig ends calculated using a correction that accounts for the fact that pairs spanning a given gap must be longer than that gap size (see Results below).
  2. Pairs of contig ends that are unambiguously linked by pairing information are “locked” together. In cases where two possible links are found, if the greater of the two estimated gap sizes is large enough to accomodate the smaller gap as well as its associated contig, the smaller gap is accepted. In order for contigs to be “locked” together they must be mutually unique extensions of each other based on pairing (in analogy to the U-U *k*-mer relationship in the contig-building step).
  3. The graph of locked contig ends is traversed to produce scaffolds which terminate when no linking information is available or the linking information does not represent a consistent, mutually unique pairing relation. A minimum number of links (paired or splinting) is required to accept a contig end connection. This threshold, 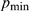, is defined by observing the distribution of the number of links per gap and may be adjusted to produce more or less conservative scaffolding. For *Pichia*, 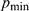 = 6 was used.
  4. Gapped contig sequence and a report of the flanking *k*-mers (“virtual primer pairs”) and the estimated size of each gap are generated and passed on to the next phase of the process, gap-resolution.
**merauder.pl.** merauder.pl closes gaps contained within scaffolds using reads that are projected to lie within the gap based on their mate reads.
  1. For each gap in the scaffolds, reads that project into the gap by direct alignment and unaligned reads whose mates' alignments suggest that they fall into the gap are collected as potential gap-fillers.
  2. Potential gap-filling reads are searched to identify those that contain both gap-flanking primer sequences and produce a closure within a given tolerance of the estimated gap size (the tolerance is based on the pair-end separation uncertainty). Such reads are said to “splint” across a gap. Note that some gaps from oNo scaffolds may be negative, indicating that the flanking contigs overlap but that the overlap is either too short or repetitive (*i.e.*, relevant *k*-mers are not in the U-U set). If splinting reads are found, then the gap is filled (or negative gap joined) if there is a unique gap-resolving sequence found in all reads that contain both primers. (Note that an optional more aggressive gap-resolution may be obtained by using the most common gap-resolving sequence and eliminating the uniqueness requirement.)
  3. If “splinting” fails, merauder.pl attempts a *k*-mer walk starting from the forward primer using the meraculous algorithm above (“mini-meraculous”) . The gap is closed if a unique path to the reverse primer is found that is within tolerance of the estimated gap size. Should the gap fail to close due to an unresolved repeat within the gap-filling read subset, the *k*-mer size is iteratively increased by two until either the gap is successfully closed or the failure is due to a lack of extension data (*i.e.*, only reaching an “X” in the graph terminates the process).
  4. Gap-resolved scaffolds are reported with gap closure sequences indicated by lower-case letters, as well as a report of the success/failure of each attempted gap resolution.

### Multiple insert sizes

The oNo and merauder steps may be iterated if multiple insert sizes exist, using paired end sets of increasing insert size.

### Lightweight Hash

To reduce the memory needed to store and randomly access the deBruijn graph, we designed and implemented a lightweight hash scheme that uses a recursive collision strategy with multiple hash functions to avoid explicitly storing the keys themselves. In the typical use case, there is a fixed dictionary of keys and associated values.

First, the hash must be “primed” as follows: (we assume there are hash functions h_0_…h_n_ already defined).

1. Initialize hash depth d to 0, write all keys to file F_d_.
2. For all keys in file F_d_, evaluate the hash function h_d_ and update a “primer object” P_d_ to keep track of which hash values occur multiple times at hash depth d (i.e. the keys collide under the hash function h_d_).
3. Write all colliding keys to file F_d+1_ ; increment hash depth d.
4. Repeat steps 1,2 until the number of colliding keys is 0.

All primers P_0_…P_d_ are then sent to the lightweight hash initializer to create a lightweight hash object. Thereafter, each key-value pair is simply added to the hash object: the hash checks the primer information to determine at which level of the recursion to store the value, while the key itself is discarded. At this point, the hash is ready to be queried. Note that the client must never attempt to look up a key that was not used in the priming step, as the hash cannot verify the identity of the key associated within a given value after priming.

### Using the lightweight hash in meraculous

In the contig generation stage, a lightweight hash object stores all relevant *k*-mers and allows contigs to be formed by walking from random “seed” starting points. Preprocessing is done to ensure that both U-U mers and terminating *k*-mers connected to those *k*-mers are stored in the hash. The terminating *k*-mers are needed because lightweight hashes do not support queries on non-existent keys. The lightweight hash is first “primed” by exposing it to each *k*-mer. Next, the *k*-mers are loaded, along with their extension codes, as key-value pairs.

### Implementation

The algorithm was implemented in a combination of C and Perl and uses SWIG to wrap the lightweight hash data structure. All benchmarks were run on 32-core AMD Opterons running at 1.8 GHz with 512 GB RAM and the “Linux AMD64-K8-SMP” operating system. At times, where noted, parallelized steps were also run on commodity clusters managed by Sun Grid Engine.

## Results

### Algorithm overview

Our algorithm follows the broad outline first described in detail for the Celera assembler [30] (see also the TIGR assembler [31]). First, we assemble contigs that do not span any repeat boundaries and therefore are either unique sequence or multi-copy sequences within recently diverged repeats. Next, we link these contigs into scaffolds, using paired-end links to jump over unassembled repetitive regions, leaving gaps whose size and flanking sequences are known. Finally, we fill intra-scaffold gaps (“captured” gaps, or “sequence-mapped” gaps) using reads whose mate pairs constrain them to lie within the gap.

Instead of computing read-read overlaps, we use the deBruijn representation of sequencing reads in terms of (overlapping) words of length *k* (“*k*-mers”) [24]. The word size *k* plays a role analogous to the minimum confidently detectable read-read overlap in alignment-based assembly [32], and is generally an empirical parameter. Larger *k* provides more specificity, but fewer *k*-mers per read, reducing the effective depth [20]. For each *k*-mer in a read, we can define its “single-base extension” in the forward direction as the *k*-mer that results by sliding the word forward by a single base. The first *k−1* bp of this extension are the same as the last *k−1* bp of the original word.

For a random sequence of length *G*, it is sufficient to use 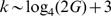, but in practice the repetitive structure of a genome can require longer *k*-mers. While this repetitive structure is typically not known *a priori*, analysis of related known genomes can suggest reasonable values of *k*. One way to assess this is to identify runs of single-base *k*-mer extensions that are unambiguous in the genome. That is, for each k-mer in a run there is only a single k-mer in the genome that overlaps it by *k*−1 bp. Such unambiguously extendable runs of *k*-mers are related to contigs, as discussed below, and we seek *k* large enough that a substantial fraction of the genome is contained in such runs. For *P. stipitis* we choose *k* = 41 to recover ∼95% of the genome in uniquely extendable *k*-mer runs longer than 500 bp. For more complex genomes like *Drosophila melanogaster*, *k* = 41 recovers ∼86% of the genome in such regions, while for the rice genome, with its long-terminal-repeat retrotransposons, *k* = 41 recovers only 59% of the genome in such regions. These runs of overlapping unique *k*-mers are a useful starting point for assembly, and can be improved using paired-end constraints as described below.

The meraculous algorithm first constructs an initial set of high confidence contig sequences by decomposing reads into overlapping *k*-mers, and identifying maximal paths in the space of all *k*-mers such that (1) every *k*-mer in a path occurs at least 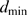 times in the dataset, (2) consecutive *k*-mers are each other's unique “high-quality” single-base extension in the read set. The *k*-mer *b* is a high quality extension of *a* if there are at least 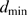 instances in the reads where *b* follows *a* (that is, the last *k*−1 bp of *a* are the same as the first *k−1* bp of *b*), and the newly added nucleotide at the end of *b* has quality at least 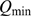. Extensions must be unique to be considered in these paths; *k*-mers that have multiple high quality extensions are candidates for the boundaries of repeats and are not included.

We mark each *k*-mer end with U if it has a unique high quality extension, F if it has more than one (is a “fork”), and X if it has no high quality extension. We then isolate the subgraph of the deBruijn graph for which all *k*-mers are designated “U-U”. By omitting forked *k*-mers, the tangled full deBruijn graph is simplified into a set of linear chains, which are easily traversed. The two parameters 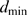 and 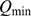 are selected empirically, as described below. Note that we make no explicit error correction; regions of reads containing errors are excluded from participating in U-U paths based on *k*-mer depth and sequence quality.

Given a set of U-U contigs, we next map reads back to these contigs by alignment. For simplicity we use BLAST, but other algorithms better suited to short-reads can be substituted, as long as alignments of reads to multiple contig locations are reported (see below). Since a *k*-mer that occurs in the U-U graph occurs only once in the U-U contigs, we require at least a *k*-bp exact match to seed the alignment of reads back to the U-U contigs, and allow mapped reads to project off the ends of contigs. Using alignment to map reads relieves us of the need to track read placements through the initial traversal of the U-U subgraph, simplifying the implementation. Once paired-end reads are placed, uncontested pair-linkages between contigs are used to form scaffolds.

Short gaps between successive contigs can then be filled in by applying the U-U procedure to the small subset of reads that are inferred to lie in a gap based on the placement of their paired end sequence. As with Sanger reads, this gap-filling process is dramatically simplified relative to the full assembly problem, since only a small region is assembled for each gap. Gap filling is readily parallelized, and can be iterated using progressively longer pairs.

### A novel lightweight hash for the deBruijn graph

It is common to store and access a deBruijn graph using a hash, which is a data structure that enables rapid lookup of a “value” associated with each “key.” To efficiently store and access the U-U deBruijn graph, we use a hash in which the “key” is a U-U *k*-mer, and the “value” is the (unique) high quality nucleotide that follows the key in the read dataset. In a conventional hash, a hash function h(key) is used to map each key into a position within a linear array of length H. The hash function is approximately uniformly distributed between 1 and H. Since multiple keys can hash to the same value, the data structure and methods must allow for such “collisions,” at additional cost in speed and memory. In a typical hash implementation, the possibility of collisions for a general and possibly changing set of keys require that keys themselves also be stored in the array.

Since the number of distinct keys is comparable to the genome size G, the memory that would naively be required to store the hash is ∼2G*(k+1) bits, with most of the memory cost associated with storing the key. (The factor of two arises from allocating two bits per nucleotide.) For example, for a human genome G∼3×10^9^; for k = 75, storing this hash would require 450 Gb. Unlike many applications of hashes, however, most of this memory is required to store the keys; the value associated with each key is only a single nucleotide (two bits). Working with such a hash requires either large memory systems [11] or distributed memory parallelization schemes [9].

To dramatically reduce the memory requirement for meraculous, we developed a novel perfect static hashing scheme that can be applied whenever the complete set of keys is known initially and does not change during the use of the hash, as is the case with the U-U deBruijn graph for a given shotgun dataset. In contrast, general dynamic hashing schemes typically retain the flexibility to add new (key, value) combinations at any time. Our hashing scheme is “perfect” in the sense that the average lookup time does not depend on the genome size. For a genome of size G, our hash requires only ∼e*G bytes of memory, independent of the choice of k, where e = 2.71828… is base of natural logarithms. The U-U hash for a human genome then requires only ∼8 Gb, a ∼60-fold memory savings relative to a standard hash and well within the range of many desktop systems.

Our perfect hash h(u) is constructed using a preprocessing step that iteratively identifies and progressively eliminates collisions for all U-U *k*-mers (Methods). Let h_i_(u) be a series of independent hash functions defined on *k*-mers. Each hash function h_i_(u) returns an integer between 1 and H_i_ that is assumed to be uniformly distributed over that range. Then a perfect hash h(u) can be defined iteratively as follows. First, compute h_1_(u) for all U-U *k*-mers, and record all collisions. Applying the Poisson distribution, H_1_*exp(−G_1_/H_1_) *k*-mers do not collide. For such k-mers, we assign a hash “level” of 1, and define the perfect hash by h(u) = h_1_(u). The G_2_ = G_1_−H_1*exp(−G_1_/H_1_) *k*-mers that collide at level 1 are then hashed at the second level using an independent hash function h_2_(u) with a reduced range H_2_. Those that do not collide are assigned h(u) = H_1_+h_2_(u); those that do collide are passed to the third level. This process is iterated until there are no more collisions.

The result is a “perfect” hash h(u) that, by construction, has no collisions. Since each of the input U-U k-mers is uniquely mapped by this function, we do not need to store the “key” k-mer with each entry, and need only store the “value,” which is just a single nucleotide. This results in a memory savings of order 1/k.

The total memory usage is H_tot_ = H_1_+H_2_+H_3_+… If for each iteration we use a hash size H_i_ proportional to the number of elements G_i_ to be hashed, i.e., H_i_ = λG_i_, then it is straightforward to show that the optimal λ = 1, and the total memory usage is H_tot_ = e*G_1_. In practice we do not allow H_i_ to drop below some cutoff H_min_∼1,000, to avoid excessive iteration. Although the maximum number of iterations (levels) needed to avoid collisions is order log(G), the average number of iterations needed is *e* in the Poisson approximation.

### *Pichia* sequencing summary, accuracy, and coverage

As a test dataset for assembling small eukaryotic genomes, we produced 87.3 million paired 75-bp reads for *P. stipitis* CBS 6054 using the Illumina GA II sequencer. Two libraries were sequenced, a ∼300 bp insert standard library (two lanes on a GAII Instrument) and a ∼3 kbp mate-pair (“jumping”) library (one GAII lane), as described in Materials and Methods. The two short-insert paired-end lanes had a somewhat higher cluster density than the mate-pair library (15.5 and 15.7 million clusters reporting sequence vs. 12.4 million). These reads yielded data that totals 6.55 Gbp, or nominal 425× redundant coverage of the 15.4 Mbp *P. stipitis* genome.

The per-base error rate relevant to *k*-mer assembly can be estimated by measuring the probability that a *k*-mer that starts at position *i* in a read (and ends at *i*+*k*−1) is observed in the genome. For the *Pichia* dataset, we find that the matching probability against the reference genome is higher for forward reads of a pair than for reverse reads. For these three lanes, the matching probability of the first 41-mer ranges from 80.9%–87.8% for forward reads, and 70.5%–77.4% for reverse reads. Similarly, the matching probability for the last 41-mer (beginning at *i* = 35 for our 75 bp reads) ranges from 72.7%–77.1% for forward reads and 54.2%–71.1% for reverse reads.

Overall, the matching probability for all 41-mers is 74.2%, so that ∼3/4 of all 41-mers are error-free. If we crudely assume that errors are uniformly distributed across reads (and neglect the effect of contamination, which also reduces the matching probability) then this corresponds to a per-base error rate of 1−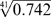 = 0.7%. In the absence of a reference genome as we have for *Pichia*, we find that Illumina quality scores provide a useful surrogate for the accuracy of base calls, so that the probability that a *k*-mer is correct is well-approximated by 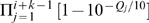, where 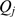 is the Phred [33] quality score at position *j* (data not shown).

Counting both strands, the *Pichia* nuclear genome contains 29,746,832 distinct 41-mers (*i.e.*, 41-bp words). 29,746,314 (99.998%) of these occur at least once in the Illumina shotgun data set. The mitochondrial genome contains 60,344 distinct 41-mers and all occur at least once in the data set. (68 distinct *k*-mers occur in both the nuclear and mitochondrial genome, and all occur in the dataset).

Due to sequencing errors, the *Pichia* shotgun data set contains 1,211,630,294 distinct 41-mers, ∼40-fold more than found in the genome. Most of the errors are single occurrences of a *k*-mer in the dataset, and are due to isolated base-calling errors. In particular, 1,042,166,572 (86%) of observed 41-mers occur only once in the data set, of which only 96 (9.2×10^−6^%) are true genomic mers. The size of the 41-mer set used in an assembly can therefore be dramatically reduced with minimal impact by discarding k-mers that occur only once in the dataset, since the vast majority of these are erroneous. The remaining ∼140 million erroneous 41-mers found in the dataset but not in the genome are recurrent sequence errors in the same sequence context (which may or may not occur in multiple locations in the genome).

### Depth statistic

**Figure 3:**
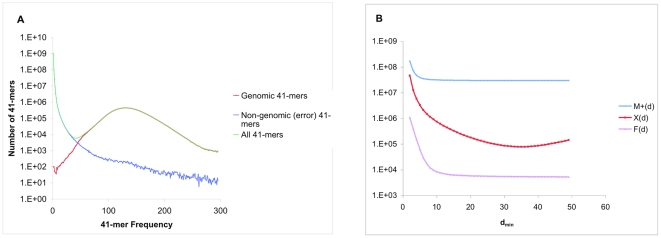
k-mer frequency and extension characteristics in *Pichia*. **A. 41-mer frequency distributions**. The overall 41-mer distribution (green) is decomposed into genomic (red) and non-genomic (yellow) contributions. At fewer than ∼30 occurrences non-genomic (error-induced) 41-mers dominate. The modal frequency is ∼135. **B. Graph features as functions of d_min_**. The total number of nodes (blue), total number of X-terminated nodes (red), and total number of F-terminated (yellow) nodes in the 41-mer graph are calculated as functions of the assembly parameter d_min_. We find the optimal assembly to occur at d_min_ = 10.

A common statistic for a sequencing project of *N* reads with average read length *R* is the raw depth of coverage *d = NR/G* = total number of nucleotides sequenced divided by genome size [32]. Assuming no errors, the number of times that a *k*-mer covers a given nucleotide in the genome is 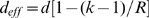, since each read of length *R* only contains *R−k*+1 *k*-mers (see, *e.g.*, [20]). This reduction in effective depth is equivalent to the 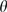 parameter introduced by Lander and Waterman in the analysis of restriction maps [32], with *k*−1 corresponding to the minimum detectable overlap between reads in the deBruijn formulation of assembly. Since *k* is comparable to the read length *R* for many short-read assembly applications, this factor can be substantial. Thus while for our *Pichia* dataset the raw depth is *d* = 425×, for *k* = 41 the finite read length correction reduces 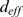 to ∼200×. A similarly large factor arises from sequencing errors; as we have seen, ∼3/4 of observed 41-mers in *Pichia* are error-free. Since ∼75% of the k-mers contained in the reads map perfectly to the genome, the effective depth of true *k*-mers is ∼150×, consistent with the mean multiplicity of 145× (modal value 130×, see **Figure 3A**). (The mitochondrial genome is at 2,900× in true 41-mer coverage.)

### Paired-end separation, chimerism, and mate-pair artifacts

To assess insert size distributions and chimerism rates independent of the assembly, we aligned reads from one lane of short insert pairs and one jumping library lane to the finished reference genome using BLAST (see Materials and Methods). The single highest scoring HSP (high-scoring segment pair [29]) was retained for each read. (In cases where multiple equally high scoring HSPs exist a best hit was chosen at random, so the chimerism rate inferred from this result should be considered an upper bound.) For the short insert lane, 11,472,868 read pairs had both ends aligned to the genome, so that ∼73% of reported clusters provide a successful read pair. The aligned pairs from each lane therefore represent ∼200× physical (“clone”) coverage of the genome. 150,085 pairs (1.3%) had best hits on differing chromosomes and 27,045 pairs (0.2%) align to the same chromosome but on the same strand. The remaining appropriately-oriented pairs have a tight, nearly symmetrical insert size distribution with mean and standard deviation of 279±7 bp (see **Figure 1A**). 174,044 of these pairs (1.5%) have ends separated by a distance more than three standard deviations above or below this mean value. We estimate from this an upper bound of 3% chimeric pairs in this library.

For the ∼3 kbp jumping library, 10,380,635 read pairs had both ends aligned to the genome, so that 84% of reported clusters provide a successful read pair. Of the aligned read pairs, 3.7% had ends hitting different chromosomes, and 0.8% hit on the same chromosome but the same strand. The remaining oppositely oriented read pairs have a bimodal distribution of separations Approximately 2/3 of all read pairs are directed away from each other and ∼3.2 kbp apart, as expected. Most of the remaining aligned, oppositely directed read pairs are directed towards each other and separated by less than 500 bp. This second group of pairs (“innies”) represents an artifact of mate pair library construction, in which the sequenced fragment is derived from a portion of the circularized DNA that does not contain the junction region (see **Figure 1B**).

The orientation and separation of these artifactual pairs makes them easy to exclude in the scaffolding step (Materials and Methods). The distribution of the innie separations is not normally distributed, and contains at least three components: a broad peak at ∼100 bp, and two somewhat narrower peaks at ∼300 bp and ∼400 bp. Excluding the “innies”, the mean and standard deviation of the end-separation for the jumping library is 3,273±196 bp, although the distribution is somewhat skewed, with mode ∼3,215 bp and half maximum range ∼3,045–3,525 bp (**Figure 1C**). Since a negligible fraction of the “innie” artifact is due to chimerism (which would be unlikely to yield paired reads within 500 bp and with a specified orientation), we can estimate the chimerism rate of mate pairs as less than ∼7%. The mate pairs provide a staggering ∼1,450× spanning coverage of the genome.

### Multiplicity distribution, error rates, and local properties of the deBruijn graph

The multiplicity of a *k*-mer is the number of times it occurs in the dataset [24], [28]. The multiplicity distribution *n*(*d*) is then the number of *k*-mers that occur exactly *d* times in the dataset. If sampling is random, and in the absence of errors, then *n*(*d*) is Poisson distributed with mean 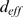. As noted previously [28], in practice *n*(*d*) has a sharp peak near *d* = 0 and another broad peak somewhat below 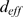. The peak near zero corresponds to *k*-mers that arise from relatively rare sequencing errors; the peak near 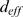 corresponds to *k*-mers that occur in the genome and are present in many reads. A simple way to distinguish erroneous *k*-mers from true *k*-mers is to separate them based on a depth cutoff *d*_min′_, retaining only *k*-mers with at least this multiplicity.

The number of U-U contigs of the deBruijn graph depends on the choice of *d*_min_ (which in our formulation determines the nodes and edges of the graph). For high values of *d*_min_, U-U contigs are likely to terminate at positions marked X, indicating that the terminal *k*-mer of the contig has no single base extensions that occur in the dataset more than *d*_min_ times. In contrast, for low values of *d*_min_, many U-U contigs will terminate at F (forked) positions where the terminal *k*-mer of the contig has two (or more) possible single base extensions, each with at least *d*_min_ occurrences in the dataset. Ideally, we would choose *d*_min_ to produce the fewest U-U contigs. We show next that the number of contigs as a function of *d*_min_ can be expressed simply in terms of *k*-mer-local properties of the deBruijn graph. This allows us to identify an appropriate choice for *d*_min_ prior to the time/memory-intensive U-U contig formation step.

The number of *k*-mers with at least *d* occurrences is given by 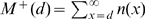, and similarly the number of *k*-mers with fewer than *d* occurrences in the dataset is 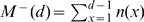. The total number of *k*-mers is simply 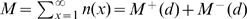. We note that *M* ^+^(*d*) is also the number of *k*-mers in the graph when *d*_min_ = *d*, and similarly *M* ^−^(*d*) is the number of *k*-mers excluded from the graph when *d*_min_ = *d*.

Let *n*_1_(*d*) and *n*_2_(*d*) be the number of *k*-mers with precisely *d* high quality extensions to their most frequent next *k*-mer, and their second most frequent next k-mer, respectively. Then 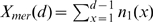 is the number of *k*-mers that are X-terminated when *d*_min_ = *d*, and 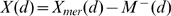 is the number of *k*-mers *in the graph* that are X-terminated when *d*_min_ = *d*. Similarly, 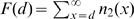 is the number of *k*-mers *in the graph* that are F-terminated when *d*_min_ = *d*. So finally, the total number of contigs when *d*_min_ = *d* can be written as 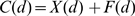, which is readily calculated from histograms that are produced by meraculous.

Results for *Pichia* with *k* = 41 are shown in **Figure 3B**. Evidently, the “X”s dominate the “F”s because of the large number of *k*-mers that arise from low frequency error. Minimizing *C(d)* would lead us to choose *d*_min_∼30. In practice, *d*_min_∼10 yields a much better assembly, which is near the “knee” in the *F*(*d*) curve. While there are more total “contigs” at this point, the great majority of them are small contigs of size ∼2*k*−1 with a central erroneous base. These small contigs are disconnected from the rest of the graph, and are discarded in the output of meraculous due to a minimum contig size cutoff ∼2*k*. Distinguishing between these small erroneous fragments and true contigs requires more than nearest-neighbor information on the graph. In practice, however, we find empirically that the best results occur for *d_min_* just above the rise in *F*(*d*).

### Scaffolding using paired-ends

Rather than tracking the position of reads through the de Bruijn graph, reads were mapped to the U-U contig set by alignment; for simplicity, BLAST was used, but other aligners designed for short reads could be used instead. As noted above, the *k*-mer uniqueness of the initial U-U contigs means that read-contig alignments with exact *k*-mer matches are necessarily unique placements of that *k*-mer. Gap filling (described below) removes this property of the contigs, since the sequences between U-U contigs need not be unique. We represent gap-filled sequence by lower case letters, which both (1) indicates the derivation of the sequence as outside of the U-U subgraph, and (2) allows us to run BLAST in a mode that prohibits seeding matches in gap-filled sequence. Reads can be (1) placed entirely within a contig, (2) project into a gap, or (3) “splint” across two contigs if the read aligned consistently to the ends of two different contigs. The splinting configuration allows a gap to be closed directly.

Paired-end sequences from sheared and size-selected ∼279-bp fragments were used to create an initial scaffolding. The pair-ends have a tight, nearly symmetrical insert size distribution (standard deviation 7 bp, see **Figure 1A**), and provided ∼400× spanning clone depth, with negligible chimerism. Typical contig-contig links involve several hundred pairs (mean = 310); scaffolds were produced using uncontested linkages from p_min_ or more read pairs, where p_min_ = 6. For the ∼3.2 kbp jumping library, the mean number of paired-end links between contigs is 809, with the weakest uncontested link is spanned by 37 pairs. (This can be substantially less than the overall depth for long gaps, since only pairs with separations from the high end of the distribution can span long gaps, see below.)

### Insert size estimation accounting for bias

The sizes of captured gaps can be estimated from spanning pairs given a known distribution of separations between paired end sequences. Accurate estimates, however, must correct for the bias introduced by the fact that the pairs that span a given gap of size *g* must be longer than *g+2R*, where *R* is the read length. Since the probability that a given read pair of separation 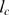 spans a gap is proportional to the size of the spanning region (the unsequenced portion of the genome between the two end-reads, 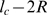), the mean separation of pairs spanning a gap of size *g* can be written as
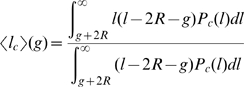
(1)where 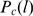 is the distribution of end separations in the library. If we model 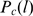 by a normal distribution with mean 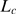 and standard deviation 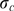, then analytic estimates can be made in the small and large gap limits. In the small gap limit 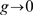,
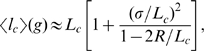
(2)while in the large gap limit 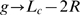


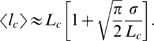
(3)The true gap size is then the self-consistent solution to
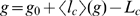
(4)where 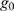 is the naive gap size (assuming the mean of the spanning pairs is the overall mean 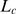). This equation can be solved iteratively. In practice, it is initially tabulated for each possible gap size.

### Closure of gaps

**Figure 4:**
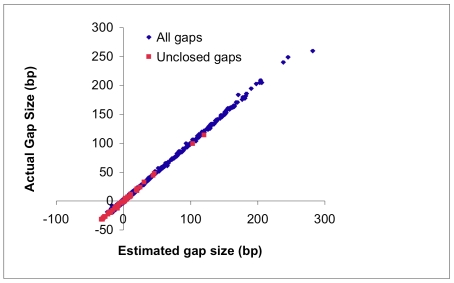
Estimated gap sizes vs. actual contig separation in the *Pichia* genome. 75% of the initial inter-contig gaps are resolved during gap closing. 97% of gaps are found to be within 4 bp of their estimated size, and 58% within 1 bp.

The estimated gap sizes that result from scaffolding the U-U contigs are shown in **Figure 4**, plotted vs. the true gap size. (The true gap size is known from the *Pichia* genome, and is shown to demonstrate accuracy of the gap size estimates; this information is not used in the assembly.) “Negative” gaps arise when adjacent U-U contigs cannot be joined in the U-U graph, but are inferred to overlap based on paired-end constraints. This situation can arise due to short repetitive sequences (typically tandem short microsatellite repeats) whose associated *k*-mers are not in the U-U set, which prevents a U-U path from linking the contigs. Nevertheless, reads can sometimes be anchored by uniquely occurring *k*-mers in the two flanking contigs. Such “splints” are only allowed if their mate pair read is placed nearby with the appropriate orientation. 95% of estimated negative gaps (938 out of 985) were closed, as were 36% of positive gaps (183 out of 515), resulting in an approximately four-fold increase in contig N50 size after gap resolution.

For each gap that is not spanned by splinting reads, we collect the reads that are projected to lie within the gap based on the location of their pair. Even if the gap contains a repetitive sequence, this modest collection of reads often has a simple assembly, since there is no interference from reads that lie in other similar copies of the repeat. To close such gaps, we attempt a meraculous assembly of the reads projected to the gap. Since in some cases short localized repeats are still present, if no path across the gap is found that agrees with the gap estimate, *k* is incremented by 2 and another attempt is made. This iterative procedure either terminates when a gap-filling path is found, or all paths connecting the flanking sequences terminate by X, indicating lack of unique continuous sequence. Using both splints and iterative meraculous assemblies, 75% of gaps between U-U contigs are closed. 97% of the gap-filling sequences are within 4 bp of the estimated gap size, and 58% are within 1 bp. Gap filling sequences are reported in lower case, since they do not have the uniqueness property of U-U contigs. Though there are no such errors in the *Pichia* assembly, we have observed rare errors occuring in gap-filled sequence due to the collapse of short tandem repeats.

### Pairing from a jumping library

A single “jumping” library was produced by shearing genomic DNA to ∼3 kbp, circularizing it, and shearing the circles again to produce short ∼250 bp fragments that were then sequenced at both ends. Nearly 70% of the paired-ends produced in this manner are oriented away from each other and separated by ∼3.2 kb on the genome, as expected. The distribution of insert sizes is slightly skewed (**Figure 1C**). The remaining ∼30% of the pairs were directed towards each other and separated by less than ∼250 bp, a configuration that results from sequencing fragments that do not include the junction of the ∼3 kbp circles (**Figure 1B**). These aberrant pairs can be excluded by requiring that only end-sequences that lie >500 bp from the end of a contig are used (**Figure 1C**). This in turn limits the order and orientation from jumping libraries to be done on contigs longer than this length scale.

### Using fosmid-ends for chromosome-scale scaffolding

We performed a long-range scaffolding using paired-end Sanger sequences from ∼9,200 fosmid clones generated previously [26] (insert size ∼36±3.2 kbp; 21.5× clone coverage). When the assembly is bolstered by this modest amount of additional long-range linking information, 90% of the genome is spanned by 12 scaffolds, all longer than 344 kbp. Since the *Pichia* genome is comprised of 8 chromosomes ranging from 980 kbp to 3.5 Mbp, the fosmid-end-scaffolded assembly therefore recovers chromosome-scale sequences.

### Accuracy of *Pichia* assembly

The meraculous assembly reconstructs 95% of the *Pichia* genome in long contigs and scaffolds. The contig N50 is 101 kbp, and the scaffold N50 is 269 kbp. (The contig N50 is the length such that half of the assembly is in contigs longer than that length; scaffold N50 is similarly defined.) When compared with the finished reference sequence, we observed no local sequence errors or global misjoins. More precisely, seven single nucleotide discrepancies were noted, but all seven loci had unanimous support for the meraculous consensus among the Illumina reads, and no support for the finished reference. These seven discrepancies represent errors in the reference sequence and not genotypic differences between the original and current projects, since the genomic DNA was from the same source. The total assembled contigs spanned 14,703,442 bp, and covered 14,763,519 bp of the reference genome, with ∼124 kbp of identically duplicated sequences in the reference genome that are assembled only once. Only 4.2% of the reference sequence was unaligned to the assembly. 20% of these missing bases occurred within the first or last 2% of chromosomes, and are telomeric sequences. Half of the missing bases are in 38 long stretches of more than 5 kbp, and 13 stretches longer than 10 kb account for about a third of the missing bases. These regions represent chromosomal regions that are typically annotated as transposable elements or repetitive genes, including the rDNA locus (See Supplemental Table S1).

### Assembly with a reduced dataset

The *Pichia* dataset described here includes two lanes of short ∼280 bp pairs, and 1 lane of medium ∼3 kbp pairs, providing a total of ∼150× sequence coverage based on the distribution 41-mer multiplicities. Assembly quality decreased only marginally when we reassembled with only a single lane of short pairs (contig N50 90 kbp; scaffold N50 254 kbp; total assembled length unchanged). With half a lane of each paired-end type (∼1/3 of total starting data, or ∼50× true 41-mer coverage), the typical contig size was halved (N50 = 41 kbp) but the N50 scaffold length decreased only slightly (250 kbp); again the total assembled length was unchanged. When only 20% of a lane of each paired-end type was included (∼13% of the starting data, or ∼10× depth based on 41-mer count), however, the contig N50 and total assembled lengths decreased substantially.

### Implementation

Most steps of the meraculous assembly pipeline are parallelized to take advantage of commodity clusters, by partitioning reads or *k*-mers among processors. Additional parallelization is possible since gap filling can be done independently for each gap; in practice, this step is fast compared with other steps. The two steps that are not parallelized are (1) the construction of the U-U subgraph, which requires the entire *k*-mer hash to be held in memory, and (2) the scaffolding step (which is not memory intensive).

### Benchmarking against other short-read assemblers

To benchmark meraculous against other short-read assemblers, we assembled a publicly available *E. coli* K-12 MG1655 dataset of 10.4 million pairs of 36-bp reads, with insert size 215±11 bp. A finished reference sequence for this 4.64 Mbp genome is available [27]. The short-read dataset represents a nominal ∼160× shotgun coverage (total sequence/genome size), although the distribution of 21-mer frequencies peaks at 65, due to both short read length (see 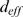 above) and errors. Assemblies of this dataset are reported in refs. [9] (for ABySS [9], EULER-SR [19], SSAKE [34], and Edena [35]), [23] (for AllPaths2 [23], as well as Velvet [20] and EULER [19]) and [11] (for SOAPdenovo). Assemblies vary depending on parametrization and other details. With parameters *k* = 21, 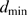 = 9, and p_min_ = 5, meraculous assembled 97.8% of the 4.64 Mbp genome into contigs ranging from 200 bp to 175 kbp, with half the assembly in 36 (26) contigs (scaffolds) longer than 40.7 (56.6) kbp. (Our assembly includes 26 contigs that are redundant on the genome, which represent perfect repeats spanning 51 kbp of the genome.) While the meraculous contigs and scaffolds are comparable in size to those produced by other assemblers on this data [9], [11], [23] no assembly errors were made (see **Table 1**). The number of errors reported for other assemblers on this dataset range from 1 for AllPaths2 to 36 for SSAKE. Four apparent discrepancies between the meraculous assembly and the reference (one insertion, one deletion, and two substitutions) were identified. In all four of these cases, Illumina reads unanimously support the meraculous sequence over the Genbank reference, suggesting either an error in the reference or a slight difference in genotype between the Sanger project and the Illumina sequence (see also ref. [23]).

**Table 1:** Comparison of assembles of *E. coli* K12 MG1655 benchmark dataset.

Assembler	Assembly as reported in	Contig N50 (kbp)	Scaffold N50 (kbp)	Coverage	Errors reported
Allpaths2	Allpaths2	337	2,680	99.3%	Base accuracy Q67; no misassemblies
Soapdenovo	Soapdenovo	89	105	NR	5 incorrect contigs
Velvet	Allpaths2	62	298	97.7	Base accuracy Q34; 6.9% of 10 kb regions missassembled
Velvet	ABySS	54	NR	98.8	9 incorrect contigs (mean size 33 kbp)
Euler-SR	ABySS	57	NR	99.8	26 incorrect contigs (mean size 52 kbp)
Euler	Allpaths2	19	19	94.6	Base accuracy Q30; 7.0% of 10 kb regions misassembled
**Meraculous**	**This report**	**41**	**57**	**97.8%**	**No errors** *
Edena	ABySS	16	NR	99.1%	6 incorrect contigs (mean size 13 kbp)
ABySS	ABySS	45	NR	99.4%	13 incorrect contigs (mean size 33 kbp)
SSAKE	ABySS	11	NR	99.99%	38 incorrect contigs (mean size 6 kbp)

In ref. [9] analysis of ABySS, Velvet, Euler-SR, SSAKE, and Edena, only contigs of at least 100 bp were considered and genome coverage was based on full length, partial, and broken alignments with at least 95% identity to reference. Contigs with broken alignments, or that aligned with less than 95% identity, were considered incorrect. In the ref. [23] analysis of Allpaths2, Velvet, and Euler, only contigs of at least 1 kbp were considered. Genome coverage computed as the fraction of 100-mers in the reference sequence that are present in the assembly, allowing for multiple occurrences in the assembly. Base quality reported as total number of discrepancies to reference, computed over ∼10 kb assembly segments that contain fewer than 1% such discrepancies. Misassemblies were reported as the total fraction of bases in ∼10 kb segments containing at least 1% error. In the ref. [11] summary of Soap denovo assembly, contigs >100 bp were reported.

NR: not reported.

*Four localized discrepancies were noted between our meraculous assembly and the E. coli K12 MG1655 reference sequence. As described in the text, further examination showed that all four discrepancies were in fact errors in the reference (or mutations in the lineages separating the MG1655 reference sample from the short read dataset sample). Analysis of errors reported for other assemblers have not been analysed.

**Figure 5:**
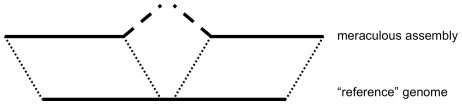
Differences between *E. coli* meraculous and reference sequence identify transposon insertion. Bottom line shows portion of the Genbank reference genome for *E. coli* str. K-12 substr. MG1655 produced by Sanger sequencing and directed finishing strain [27]. Top shows alignment of the *de novo* meraculus contigs to reference sequence. Solid lines agree perfectly. Angled dashed lines represent unaligned meraculous contig-ends that correspond to the beginning and end of a transposable element. All short-read data supports the meraculous sequence, indicating either insertion of the transposon in the Illumina-sequenced lineage, or an error in the MG1655 reference.

We also identified three locations in the finished reference sequence (∼257,905, ∼1,298,720, and 1,871,060) that were discrepant in a manner consistent with the insertion of an IS1 transposase in the meraculous assembly relative to the reference. These have not been noted previously in other Illumina assemblies of this dataset. The situation is shown schematically in **Figure 5**. At these locations, the meraculous assembly is confirmed by all available Illumina data, which does not match the reference sequence. We suggest that these loci are either incorrectly finished regions (which seems unlikely given the special care used in [27], who were focusing on intraspecies variation) or, more intriguingly, recent insertions of IS1 in the lineage separating the *E. coli* K-12 MG1655 genotype used by [27] from the sample used in Illumina library construction.

### Comparison of meraculous *Pichia* assembly with other short-read assemblers

We applied several previously published short-read assemblers to the *Pichia* dataset, with results summarized in **Tables 2** **,** **3**. Details of the assembly protocols and resource utilization of the assemblers used in this comparison are included in Supplemental Text S2. Compared with the other assemblers tested, meraculous has the fewest errors (none in the genome, vs. ∼1/10 kb for the others), and comparable completeness (∼95%), contig, and scaffold N50. (Although ABySS has substantially more total assembly than meraculous and the other assemblers that were tested, a large fraction of the additional ABySS sequence is redundantly assembled, which explains why the unique coverage is less than that of the others (last column of Table 3).)

**Table 2:** Comparison of *P. Stipitis* assembly scaffold characteristics (including scaffolds of size at least 2 kbp).

Assembler	No. Scaffolds	Total Size (Mbp)	Scaffold N50 (no. / kbp)	Total gap bases (kbp; %)	Scaffolding errors
ABySS	111	15.48	20 / 263	7.3 (0.05%)	0
Meraculous	118	14.79	18 / 269	81.7 (0.55%)	0
SOAPdenovo	88	14.74	14 / 348	156 (1.06%)	0
Velvet	157	14.82	24 / 202	136 (0.92%)	78

To assess accuracy of the assemblies, contigs were aligned to the reference genome using BLAST. Scaffolding errors include non-colinear arrangements of contigs within scaffolds with respect to the reference sequence.

**Table 3:** Comparison of *P. Stipitis* assembly contig characteristics (including contigs of at least 100 bp).

Assembler	No. Contigs	Total Size (Mbp)	Contig N50 (no. / kbp)	Contig error rate	Reference coverage	Unique coverage
ABySS	132	15.48	21 / 263	1/29 kbp	97.8%	92.2%
Meraculous	489	14.70	44 / 101	<1/15000 kbp	95.8%	95.8%
SOAPdenovo	561	14.58	64 / 65	1/6.4 kbp	95.2%	95.1%
Velvet	572	14.69	87 / 53	1/15 kbp	96.5%	95.4%

Contig error rate is measured for only the single best-aligning BLAST HSP per contig. Reference coverage is based on the total number of bases spanned by at least one HSP; unique coverage is based on the total number of reference bases spanned by exactly one HSP.

### Simulated assembly and scaling for larger genomes

To assess the feasibility of using meraculous to assemble larger genomes, we performed two experiments with simulated data for the ∼119 Mbp genome of *A. thaliana*, which is ∼8-fold larger than the *P. stipitis* genome. First, we assembled an idealized 41-mer dataset (all 41-mers present in the TAIR8 *A. thaliana* reference). 35,208 contigs longer than 200 bp were produced, totalling 105,782,921 bp (89% of the 118,960,067 bp in the finished *A. thaliana* reference sequence). The N50 was 13.1 kb, and no errors were made. Of the 35,208 gaps between these contigs, 15,591 (44%) are negative, corresponding to short repetitive sequences that should be closed using splinting reads. Another 5,902 gaps (17%) are between 0 and 100 bp, readily captured and closed by short-insert pairs as described here for *Pichia*. These results suggest that ∼50–60% of gaps could be closed with short-insert pairs, reaching a contig N50 of ∼25–30 kbp. Only 1,302 gaps are longer than 2 kbp, further suggesting that scaffolding with medium insert pairs as described for *Pichia* would produce typical scaffolds of ∼100 kbp.

We also simulated a 100× nominal depth coverage sampling of *A. thaliana* with realistic error profiles (Methods), with 79,456,596 75-bp read pairs with end-separation normally distributed with mean and standard deviation 300±30 bp. The initial contigs (prior to gap closing) closely matched expectation based on the idealized 41-mer dataset described above (total length 105.4 Mbp; 36,854 contigs ranging in size from 200 to 102,310 bp; half the assembly in 2,375 contigs of at least 11,621 bp). With gap closing, we obtained 17,609 contigs ranging in size from 200 to 180,022 bp, with half the assembly in 1,066 contigs of at least 26,949 bp, again as expected. Scaffolding with these 300 bp pairs was modest, with half the assembly in 679 scaffolds longer 42,556 bp, consistent with estimates based on the idealized data set. This assembly contains eight localized sequence errors and one non-local scaffolding error relative to the reference sequence.

To demonstrate the memory scaling of our algorithm for larger genomes, we determined the U-U contigs for the human genome, based on a shred of the 2.8 Gbp reference sequence into its constituent 75-mers. The U-U contigs longer than 150 bp accounted for 98% of the reference genome, with N50 contig length of 8.7 kbp. No scaffolding or gap closing step was attempted in this demonstration. As expected, only 8.8 Gb of memory was required to represent the U-U deBruijn sub-graph using our lightweight hash scheme.

## Discussion

Using meraculous, a new short-read assembler, we have shown that high quality, near-complete *de novo* assemblies of small fungal genomes can be produced using deep short-read paired-end datasets. Half the genome assembly is contained in contigs of at least 101 kbp (N50 contig), and in scaffolds of at least 269 kbp (N50 scaffold). Adding a modest number of fosmid-ends allows recovery of entire chromosomes. Approximately 4.2% of the genome (650 kbp out of 15.4 Mbp) is not captured in the assembly, representing repetitive sequences, notably including telomeric sequences, long retrotransposons, and high copy tandemly-arrayed elements. Comparing the assembly consensus to the previously finished and validated reference sequence, we find no errors across the entire assembly.

Our algorithm incorporates elements used in other long- and short-read paired-end assemblers, in a new combination and with new parallel implementations and heuristics based on our analysis of the *Pichia* dataset. The deBruijn graph, first applied to shotgun sequence assembly nearly a decade ago by Pevzner *et al.* [24] (following previous introduction in sequencing by hybridization [36]; see also [37], [38]), is the basis for all of the current generation of short-read assemblers [18]. In our approach, however, we do not construct the full de Bruijn graph defined by the reads. Instead, we limit ourselves to the “U-U” subgraph that includes only likely *k*-mers from the genome that possess unique, reciprocal, high quality extensions at each end. In this way we remove most error-containing *k*-mers and produce a graph that consists of a collection of simple unbranched paths. These paths are closely related to the “unitigs” of the Celera Assembler [30] and the “unipaths” of ALLPATHS [22] in that they represent genomic regions whose assembly into contigs is uncontested based on read-read alignments or their equivalent in the deBruijn formulation. A related approach is taken in SOAPdenovo [11]. The U-U subgraph can be readily produced with a memory footprint that scales linearly with the genome size, a characteristic of de Bruijn graph based methods.

Overall, memory usage in Meraculous depends not only on the size of the U-U subgraph, but also on the parallelization parameters used in the stages that preprocess the U-U subgraph. By dividing the k-mer sample space into disparate chunks, peak RAM usage and running time can be adjusted to user requirements. For instance, on our 32-core test machine, one can optimize for speed by allowing all *k*-mer sample chunks to be processed simultaneously: in this case, the *Pichia* assembly runs in 3 hours 37 minutes with a peak RAM footprint of 153 Gb. By varying the number of simultaneously-processed chunks processed on a per-stage basis, one can optimize for RAM use: the *Pichia* assembly then runs in 12 hours 28 minutes but with a peak RAM footprint of 7.72 Gb. In general, given *P* chunks preprocessed simultaneously out of *C* total chunks of the *k*-mer space of *M* mers and genome size *G*, the peak RAM *R* is characterized by R = O(P * M/C)+3.7 * G. In other words, meraculous can be made to fit (at the expense of increased runtime) into an arbitrarily small RAM footprint down to the limit of the U-U subgraph hash itself which, in practice, requires ∼3.7 bytes per base in the genome to store.

Our implementation avoids explicit error correction [24], [28], a feature of most other short-read deBruijn assemblers [9], [11], [19], [20], [22], in favor of a brute force approach that is made possible by the quality and quantity of current Illumina data. Error correction takes advantage of the preponderance of accurate sequence to identify outliers (*e.g.*, error-containing *k*-mers that occur only a few times in the dataset when the typical true *k*-mer from that genomic region occurs dozens or hundreds of times). Assuming that such *k*-mers contain errors, the error-correction approach seeks the minimal sequence change to convert these outlying *k*-mers into sequences found more often in the data [24]. While this approach is clearly feasible in uniquely assemblable regions of strong coverage, it is also not necessary there, since the correct assembly will often be evident anyway due to overwhelming depth of accurate sequence. From this perspective, it is sufficient to simply ignore the erroneous *k*-mer, as we do here. Our algorithm identifies these outliers (using a combined quality and depth filter) and disregards them in a robust way that does not degrade the assembly but allows the algorithms and their implementation to be simplified and streamlined.

Using mate-pair information, scaffolds of nominally single copy sequences can be constructed. Gaps captured within these scaffolds (comprising repeats) can then be back-filled using paired-ends, as first described in [16] and robustly implemented for large-scale assembly in the Celera Assembler [30]. This “gap-filling” step allows residual errors to be corrected through the construction of consensus sequences. Thus by combining the efficient deBruijn approach for determining an initial set of contigs, with a read-based approach using mate-pairs to link across and fill in gaps between the initial contigs, meraculous can produce accurate assemblies of short-read datasets.

A limitation of the current meraculous algorithm is that it assumes data from a haploid genome. In a diploid sample, heterozygous single nucleotide variations generate forks in the deBruijn graph, and our algorithm's reliance on the linear U-U component of the graph as a starting point for making contigs must be augmented to allow for bubbles in the graph that arise from such heterozygous regions.

## Supporting Information

Table S1 **Summary of unassembled genome sequences.** This table lists the locations, sizes, and annotations of 38 regions of the *Pichia* genome larger than 5 kb which contain 62% of the sequence missing from the meraculous assembly.
(DOC)

Text S1
**Optimal Choice of** 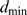 **.** This note presents a formal calculation of the contig-number minimizing choice of the assembly parameter *d_min_*.(DOC)

Text S2 **Timing and memory comparisons with other assemblers.** This note details the protocols and computational resources we used to perform assemblies of *Pichia* with alternative available assembler software.
(DOC)
