# Supplementary material for: Meraculous: De Novo Genome Assembly with Short Paired-End Reads
Source: PLoS One. 2011 Aug 18;6(8):e23501. doi: 10.1371/journal.pone.0023501 (PMC3158087; doi:10.1371/journal.pone.0023501)
Supplement: Table S1 — Summary of unassembled genome sequences. This table lists the locations, sizes, and annotations of 38 regions of the Pichia genome larger than 5 kb which contain 62% of the sequence missing from the meraculous assembly. (DOC) [file pone.0023501.s001.doc]

**Supplemental Table S1.**  **Summary of unassembled genome sequences**. 62% of missing bases in the meraculous assembly of Pichia are contained in 38 regions longer than 5 kb.  This table shows the locations, sizes, and annotations of these regions, which include telomeric DEAD-like helicases;   Zorro L1-like non-LTR retrotransposon; Ty5-like retrotransposon; tandem repetitive arrays; and a near-identical two-copy beta glucosidase.

| chrom ID | start-stop | length | annotation |
| --- | --- | --- | --- |
| chr_1.1 | 1-8,155 | 8.2kb | DEAD-like helicase (telomeric) |
| chr_1.1 | 8,776-16,860 | 8.1kb | DEAD-like helicase (telomeric) |
| chr_4.1 | 1-8,723 | 8.7kb | DEAD-like helicase (telomeric) |
| chr_7.1 | 1-11,058 | 11.1kb | DEAD-like helicase (telomeric) |
| chr_7.1 | 1,106,260-1,114,415 | 8.2kb | DEAD-like helicase (telomeric) |
| chr_8.1 | 971,156-979,380 | 8.2kb | DEAD-like helicase (telomeric) |
| chr_1.1 | 433,581-440,252 | 6.7kb | Polyprotein L1-like non-LTR retrotransposon Zorro [Candida] |
| chr_1.1 | 1,660,593-1,668,237 | 7.6kb | Polyprotein L1-like non-LTR retrotransposon Zorro [Candida] |
| chr_1.1 | 1,714,075-1,719,555 | 5.5kb | Polyprotein L1-like non-LTR retrotransposon Zorro [Candida] |
| chr_1.1 | 1,780,129-1,786,989 | 6.9kb | Polyprotein L1-like non-LTR retrotransposon Zorro [Candida] |
| chr_1.1 | 1,901,111-1,907,939 | 6.8kb | Polyprotein L1-like non-LTR retrotransposon Zorro [Candida] |
| chr_2.1 | 931,139-940,161 | 9.0kb | Polyprotein L1-like non-LTR retrotransposon Zorro [Candida] |
| chr_2.1 | 2,112,216-2,118,847 | 6.6kb | Polyprotein L1-like non-LTR retrotransposon Zorro [Candida] |
| chr_2.1 | 2,592,668-2,597,804 | 5.1kb | Polyprotein L1-like non-LTR retrotransposon Zorro [Candida] |
| chr_3.1 | 459,644-466,306 | 6.7kb | Polyprotein L1-like non-LTR retrotransposon Zorro [Candida] |
| chr_3.1 | 602,522-609,330 | 6.8kb | Polyprotein L1-like non-LTR retrotransposon Zorro [Candida] |
| chr_3.1 | 1,383,796-1,390,728 | 6.9kb | Polyprotein L1-like non-LTR retrotransposon Zorro [Candida] |
| chr_3.1 | 1,704,841-1,722,550 | 17.7kb | rDNA operon + Polyprotein L1-like non-LTR retrotransposon Zorro [Candida] |
| chr_4.1 | 274,100-286,614 | 12.5kb (2copy) | Polyprotein L1-like non-LTR retrotransposon Zorro [Candida] |
| chr_5.1 | 1,370,505-1,377,227 | 6.7kb | Polyprotein L1-like non-LTR retrotransposon Zorro [Candida] |
| chr_1.2 | 84,789-114,565 | 29.8kb | Ty5-like Retrotransposon polyprotein [Candida] |
| chr_2.1 | 1,669,998-1,704,019 | 34.0kb | Ty5-like Retrotransposon polyprotein [Candida] |
| chr_3.1 | 1,419,264-1,442,092 | 22.8kb | Ty5-like Retrotransposon polyprotein [Candida] |
| chr_3.1 | 1,442,651-1,452,230 | 9.6kb | Ty5-like Retrotransposon polyprotein [Candida] |
| chr_4.1 | 1,032,738-1,062,620 | 29.9kb | Ty5-like Retrotransposon polyprotein [Candida] |
| chr_5.1 | 646,479-666,746 | 20.3kb | Ty5-like Retrotransposon polyprotein [Candida] |
| chr_6.1 | 891,281-915,737 | 24.5kb | Ty5-like Retrotransposon polyprotein [Candida] |
| chr_7.1 | 254,910-276,429 | 21.5kb | Ty5-like Retrotransposon polyprotein [Candida] |
| chr_7.1 | 276,988-296,948 | 20.0kb | Ty5-like Retrotransposon polyprotein [Candida] |
| chr_8.1 | 285,849-326,849 | 41.0kb (2copy) | Ty5-like Retrotransposon polyprotein [Candida] |
| chr_1.2 | 1,302,321-1,309,486 | 7.2kb | 147bp x 30 + 114bp x 13 tandem array |
| chr_3.1 | 15,087-20,242 | 5.2kb | 114bp x 12 + 147bp x 20 tandem array |
| chr_2.1 | 307,130-313,583 | 6.5kb | 108bp x 60 tandem array |
| chr_6.1 | 1,689,039-1,694,489 | 5.5kb | 135bp x 18 + 132bp x 19 tandem array |
| chr_7.1 | 1,001,988-1,008,049 | 6.1kb | 135bp x 20 tandem array |
| chr_8.1 | 948,440-959,197 | 10.8kb | 126bp x 70 + 141bp x 8 tandem array |
| chr_4.1 | 1,775,707-1,782,934 | 7.2kb | beta-glucosidase (98-99% nt identical to below) |
| chr_6.1 | 1,708,452-1,715,563 | 7.1kb | beta-glucosidase (98-99% nt identical to above) |
|  |  |  |  |
