# Supplementary material for: Meraculous: De Novo Genome Assembly with Short Paired-End Reads
Source: PLoS One. 2011 Aug 18;6(8):e23501. doi: 10.1371/journal.pone.0023501 (PMC3158087; doi:10.1371/journal.pone.0023501)
Supplement: Text S1 — Optimal Choice of . This note presents a formal calculation of the contig-number minimizing choice of the assembly parameter dmin. (DOC) [file pone.0023501.s002.doc]

**Supplemental Text S1.** **Optimal Choice of $d_{\text{min}}$.**

Instead of an error correction step as performed in other short-read assemblers, we simplify the deBruijn graph by discarding *k*-mers that occur fewer than $d_{\text{min}}$times in the dataset.  Since the error rate is small, and the depth of coverage so high, this has only a small effect on our ability to assemble.  If $d_{\text{min}}$is chosen too small, however, many contigs will end at forks (*i.e.*, *k*-mer ends marked F) for which one or more branches are due to errors.  Conversely, if $d_{\text{min}}$is chosen too large, contigs will end at regions of low coverage (*i.e.*, *k*-mer ends marked X).  With some simple assumptions, we can derive an optimal choice of $d_{\text{min}}$that minimizes the number of contigs. 
Let the number of *k*-mers of frequency *x* be denoted by *n*(*x*).  We assume that *n*(*x*) can be decomposed into the sum of two contributions, the true genomic *k*-mers of frequency *x*, *t*(*x*), and the erroneous (false) *k*-mers of this frequency,  *f*(*x*).  Thus *n*(*x*) = *t*(*x*)+*f*(*x*).   We may then define the following integrals (assuming that the functions of the discrete variable *x* are smooth):

*T* = total number of true *k*-mers = $\int t(x)\text{dx}$                           (eq. S1)

*F* = total number of erroneous *k*-mers = $\int f(x)\text{dx}$                 (eq. S2)

For a given choice of $d_{\text{min}}$, the number of contig ends (*i.e.*, twice the number of contigs) that are produced will be the sum of two contributions:  those contigs $C_{T}$ that are prematurely truncated at true *k*-mers whose frequency is less than$d_{\text{min}}$ (contigs ending with X), and those contigs $C_{F}$ that are prematurely truncated at erroneous *k*-mers whose frequency is greater than$d_{\text{min}}$(contigs ending with F).  In our simple model, these values are approximated by the integrals:

                                               (eq. S3)
                        (eq. S4)

To minimize the number of contigs, we minimize the sum of these contributions $C(d)=C_{T}(d)+C_{F}(d)$, which can be rewritten using Eqs. S3 and S4 as

                                      (eq. S5)

which is extremal with respect to $d$ when the integrand vanishes.   The optimal choice of $d_{\text{min}}$ is therefore the frequency at which the number of false *k*-mers is equal to the number of true *k*-mers .  In practice, for a given observed mer-frequency distribution this value can be obtained by fitting the low-frequency *k*-mer distribution (*e.g.*, to a power law) and the peak-frequency distribution (*e.g.*, to a Gaussian) independently and finding the intersection point of the two fits.  Due to the sharp crossing of true and false *k*-mers that is typically observed, the common choice of the minimum of the *k*-mer frequency distribution [17] may be a simple and useful approximation, but is distinct from the condition derived here. As discussed in the main text, in practice a lower choice of $d_{\text{min}}$is preferred since the calculation presented here includes short contigs of length 2*k*-1 centered on errors.
