# Supplementary material for: Meraculous: De Novo Genome Assembly with Short Paired-End Reads
Source: PLoS One. 2011 Aug 18;6(8):e23501. doi: 10.1371/journal.pone.0023501 (PMC3158087; doi:10.1371/journal.pone.0023501)
Supplement: Text S2 — Timing and memory comparisons with other assemblers. This note details the protocols and computational resources we used to perform assemblies of Pichia with alternative available assembler software. (DOC) [file pone.0023501.s003.doc]

**Supplemental Text S2**: Timing and memory comparisons with other assemblers for *Pichia*.

For comparison with the meraculous assembly of *Pichia*, we used three other short read assemblers (SOAPdenovo, Abyss, Velvet). We could not use the current AllPaths-LG since the data in hand did not meet the requirements of overlapping paired-end reads. For these analyses and comparisons with meraculous, we used a Quad-Core AMD Opteron(tm) 8376 HE, with 8 CPU per core running at 2.3 GHz, and 517 GB total RAM.

**SOAP De Novo (Version 1.3, Released Nov 23, 2009)**

All libraries were used in the contig building step, while short-insert libraries were ranked 1 during scaffolding, followed by long insert lib, ranked 2.

The following steps were perfomed:

**Pregraph building**: SOAPdenovo pregraph -p 32 -K 31 -d 9

**Contig building:** SOAPdenovo contig -M 1 -D 1 -R no

**Map reads to contigs:** SOAPdenovo map -p 32

**Scaffold building:** SOAPdenovo scaff -G 50 -p 32 -L 100

Total wall clock time used was 2,360 sec (0.7 hrs), and 20.8 GB main memory was required.

**ABySS (Version: 1.2.3)**

**abyss-pe -j3 k=31 n=6  lib='lib1 lib2 lib3'**

**Memory and Time Usage:**

Total wall clock time used was 21,729 sec (6.0 hrs), and 19.5 GB main memory was required.

**Velvet (Version: 1.0.13)**

To incorporate jumping libraries, reads were reverse complemented prior to use in Velvet. These pairs were used with the longPaired option. Total wall clock time used was 16,598 sec (4.6 hrs) and 32.0 GB main memory was required.

**velveth 41** -longPaired ‘3kb long insert lib’

**velvetg -exp_cov auto -ins_length 279 -ins_length_sd 50 -ins_length2 279 -ins_length_sd 50 -ins_length_long 3260 -ins_length_long_sd 450 -min_contig_lgth 100 -min_pair_count 6**
